# Drivers of abundance and spatial distribution of reef-associated sharks in an isolated atoll reef system

**DOI:** 10.1371/journal.pone.0177374

**Published:** 2017-05-31

**Authors:** David M. Tickler, Tom B. Letessier, Heather J. Koldewey, Jessica J. Meeuwig

**Affiliations:** 1 Oceans Institute: Centre for Marine Futures, University of Western Australia, 35 Stirling Highway, Crawley, Perth, WA, Australia; 2 Zoological Society of London, Regents Park, London, United Kingdom; 3 Centre for Ecology & Conservation, University of Exeter, Cornwall Campus, United Kingdom; Leibniz Center for Tropical Marine Ecology, GERMANY

## Abstract

We investigated drivers of reef shark demography across a large and isolated marine protected area, the British Indian Ocean Territory Marine Reserve, using stereo baited remote underwater video systems. We modelled shark abundance against biotic and abiotic variables at 35 sites across the reserve and found that the biomass of low trophic order fish (specifically planktivores) had the greatest effect on shark abundance, although models also included habitat variables (depth, coral cover and site type). There was significant variation in the composition of the shark assemblage at different atolls within the reserve. In particular, the deepest habitat sampled (a seamount at 70-80m visited for the first time in this study) recorded large numbers of scalloped hammerhead sharks (*Sphyrna lewini*) not observed elsewhere. Size structure of the most abundant and common species, grey reef sharks (*Carcharhinus amblyrhynchos*), varied with location. Individuals at an isolated bank were 30% smaller than those at the main atolls, with size structure significantly biased towards the size range for young of year (YOY). The 18 individuals judged to be YOY represented the offspring of between four and six females, so, whilst inconclusive, these data suggest the possible use of a common pupping site by grey reef sharks. The importance of low trophic order fish biomass (i.e. potential prey) in predicting spatial variation in shark abundance is consistent with other studies both in marine and terrestrial systems which suggest that prey availability may be a more important predictor of predator distribution than habitat suitability. This result supports the need for ecosystem level rather than species-specific conservation measures to support shark recovery. The observed spatial partitioning amongst sites for species and life-stages also implies the need to include a diversity of habitats and reef types within a protected area for adequate protection of reef-associated shark assemblages.

## Introduction

Top predators, occupying the highest position in food webs, are thought to exert significant influence on the structure and function of ecosystems. Studies of predator-prey interactions in terrestrial ecosystems have shown that the removal and subsequent reintroduction of top level, or apex, predators has resulted in large cascading changes in animal and plant community composition [1–3]. While marine food webs may differ from those on land, with much of the primary production deriving from microscopic phytoplankton rather than sessile plants [4] and relatively high degrees of omnivory [5], similar patterns of ‘trophic cascade’ also appear to occur in marine ecosystems [6]. It is hypothesised that top marine predators influence community structure in their host ecosystems both directly, through the mortality they inflict on their immediate prey, and through indirect effects resulting from behavioural changes in prey and competitors (so called ‘fear effects’). This in turn may impact the abundance and ecological function of species at the lowest levels in the food web [7,8]. Such interactions may ultimately lead to important changes to marine ecosystem function, productivity and socio-economic value [9,10].

The role of predators in regulating community structure, and thus maintaining ecosystem function, may be of particular importance in coral reef ecosystems. Reefs support the highest diversity of fishes in the ocean [11] and over half a billion people depend directly on the services they provide, including fisheries, tourism and coastal protection [12]. Their importance is matched by their vulnerability, as reefs are now affected by a multitude of natural and anthropogenic stressors, often interacting in a synergistic manner [13]. Atmospheric and oceanic warming can amplify natural climate cycles (e.g. El Niño) leading to more frequent and severe coral bleaching events [14]. The damage to reefs from such events may then be compounded by pulse disturbances like damaging storms [15], and in many coastal areas eutrophication from agriculture and other land-based activities promotes algal growth on damaged reefs that further impairs coral recovery [16]. Recent studies suggest that reef community composition influences the resilience of reefs to many of these stresses, for example through the role that teleost and invertebrate grazers play in maintaining controlling algal growth on reef substrate following influxes of nutrients from coastal flooding [17].

On coral reefs, sharks are considered to be an important predator group connecting lagoons to reef habitats and offshore ecosystems, and exerting predation influence over a range of taxa [18,19]. Reef shark assemblages include both reef-dependent species such as grey reef (*Carcharhinus amblyrhynchos*), black tip (*Carcharhinus melanopterus*) and white tip reef sharks (*Triaenodon obesus*), and more transient but reef-associated species such as silvertip (*Carcharhinus albimarginatus*), tiger (*Galeocerdo cuvier*) and hammerhead sharks (*Sphyrna* spp.) [19]. Many reef and reef-associated sharks exhibit k-selected life history traits–relatively low reproductive rates (5–15 pups per female), late maturity (7–15 years) and slow growth–and thus high sensitivity to mortality from fishing [20]. Sharp declines in shark abundances have been observed around the world in both pelagic and coastal ecosystems, with up to 90% reductions reported for some reef species [21,22]. These losses have been linked to changes in the composition of teleost communities on reefs, in particular the abundance of grazing species, which may have impacted reef resilience [7]. While recent studies and reviews have suggested that some smaller reef shark species might be better classified as meso-predatory rather than apex species on reefs [19,23,24], their larger home ranges relative to other meso-predators (e.g. serranids, lutjanids and lethrinids) [25–28], relatively large body size [29], and broad diets [19] mean they may yet perform significant roles as top predators and as dominant intra-guild competitors. Barley et al. [30] found evidence that larger reef sharks in the Rowley Shoals may influence fish biomass, trophic structure and species richness, as well as smaller competitor shark species. Measures to support the recovery of reef shark populations are therefore likely to contribute to improving and sustaining the health and resilience of coral reef ecosystems by helping to restore balance to reef communities. Additionally, reef sharks may deliver direct economic benefits to coastal communities through their importance for dive tourism [31].

Species-specific or fisheries input and output controls [32] are widely used to reduce pressure on individual populations of marine species, but increasingly marine protected areas (MPAs) have been proposed to substantially limit all extractive activities at the ecosystem scale, protecting both reefs and the species associated with them [33,34]. Closing large areas to fishing and other extractive activities is a decision involving substantial political and economic commitment, and careful design of MPAs is required if they are to avoid failing to achieve their conservation objectives [35,36], for example through failure to adequately protect the home range of the species of interest [37]. In the case of sharks, an understanding of the natural drivers of spatial variation in shark abundance and demography at the assemblage and species level may help ensure that reserve design takes account of variations in habitat preferences by species and ontogeny [38–40].

**Fig 1:**
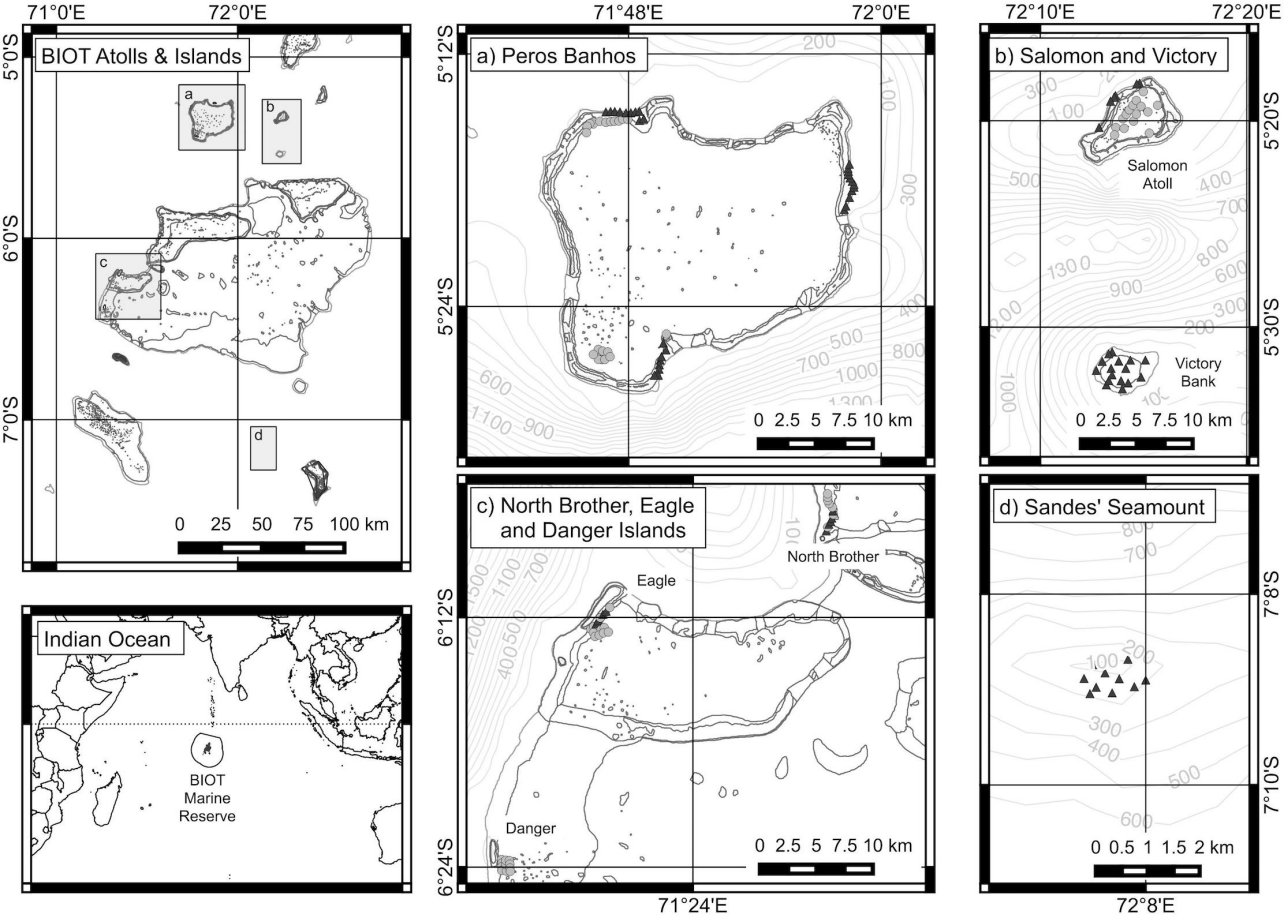
Maps of BIOT showing areas sampled with stereo-BRUVS. Panels a) to d) show locations of individual samples on reefs (black triangles) and lagoons (grey circles).

Examining the natural drivers of shark abundance ideally requires data from populations that have been minimally impacted by external factors, in particular fishing, that might artificially structure their abundance, size and distribution. While decades of marine exploitation mean that few, if any, parts of the ocean may now qualify as truly pristine [41,42], areas remote from human populations and the loci of fishing effort offer the possibility of observing reefs under near-natural conditions with relatively intact shark and fish assemblages [43]. One such candidate system is the British Indian Ocean Territory (BIOT) Marine Reserve (hence BMR), a large archipelago of atoll-based reefs in the central Indian Ocean, extending 330km north to south and 210 km east to west, which is administered by the United Kingdom (Fig 1). The northern-most reefs are 450km from their nearest neighbour, Addu Atoll in the southern Maldives, and the no-take marine reserve surrounding the reefs extends up to 370 km offshore, to the limit of the UK exclusive economic zone [44]. Prior to the declaration of the reserve, reef-based fisheries were limited to small-scale artisanal fisheries based out of Mauritius that reported only low levels of shark bycatch [45]. Illegal fishing is, however, believed to have had some historical impact on shark numbers [46]. A review of trends in diver sightings of sharks between 1978 and 2006 reported a decline in sightings of reef sharks in the BMR over that period, attributed to illegal fishing [47]. Although Graham et al. [47] estimated a decline of 90% in shark numbers based on sightings, potential biases associated with underwater visual census (UVC) methods [48,49] and the low temporal resolution of the surveys mean that part of the observed differences between survey years may have been a result of both diver-avoidance by sharks and temporal or spatial fluctuations in shark abundance at the locations surveyed, rather than reflecting overall shark abundance in the BMR. Comparisons of coral health and the overall biomass of fish assemblages between the BMR and unprotected locations show that it remains one of healthiest reef systems in the world [44,50], and therefore one of a handful of locations in which we might study reef sharks with a view to better understanding natural drivers of their distribution and abundance at an ecosystem scale.

We used stereo baited remote underwater video system (stereo-BRUVS) data collected in the BMR in February 2012 to model relationships between measures of the shark assemblage and a combination of environmental variables and measures of reef fish abundance, biomass and diversity. Stereo-BRUVS were chosen as they offer a non-extractive means of assessing shark abundance that avoids the depth limits and potential observer bias of UVC, and also allow accurate estimation of the individual lengths of animals that can be used to make inferences about size structure and to estimate biomass. We modelled shark abundance relative to environmental and biological variables. Sample depth, macro-habitat type (reef vs. lagoon), and benthic cover were used as environmental variables as sharks are known to exploit a variety of horizontal and vertical niches, potentially for competition avoidance and to exploit different prey sources [34,38,40,51–53]. Biological variables were measures of fish biomass by functional group (a proxy for prey availability), since studies of terrestrial and marine predators have found that prey availability may be a stronger predictor of mobile predator abundance or presence than environmental variables [54–57]. Functional groups included lower trophic order fishes that could be considered potential prey for reef sharks: meso-predatory piscivores with trophic level (TL) below 3.5 (e.g. labrids), planktivores (e.g. caesionids), corallivores (e.g. chaetodontids), detritivores (e.g. acanthurids) and herbivores (e.g. scarine labrids). Testing hypotheses about the drivers of spatial variation in reef shark abundance at the scale of an entire reef system is necessary to account for habitat preferences in the design of spatial protection regimes for marine conservation, and is increasingly important as the number of very large marine protected areas grows [58].

## Materials and methods

Data were collected in the BMR, a large coral reef system of over 60 individual islands grouped into seven main atolls (Fig 1). The islands, reefs and lagoons lie in the central Indian Ocean between 04°54' to 07°39' south latitude and 70°14' to 72°37' east longitude. Data were collected using stereo-BRUVS [59] over three weeks during February and March 2012. The fieldwork was authorised by the United Kingdom Foreign and Commonwealth Office and was undertaken under ethics approval from the University of Western Australia.

The stereo-BRUVS used Sony HandyCam HX7/HX12 cameras, with autofocus set to ‘infinity’ and field of view set to ‘wide’, with standard rig geometry and deployment protocols (see [60]). Reef and lagoon habitats were sampled across two atolls and a submerged reef in the north (Salomon, Peros Banhos and Victory Bank respectively), along the western edge of the Great Chagos Bank (North Brother, Eagle and Danger Islands), and at a seamount in the south of the area (Sandes Seamount) (Fig 1). Samples were collected at 35 sites that reflected a range of depths (10-80m) and coral cover (0–100%) (Table 1). An average of 4 (range 3 to 5) sampling stations were used at each location for a total of 138 samples, and each stereo-BRUVS unit was left to record for a minimum of 60 minutes.

**Table 1:** Effort, depth and habitat composition for stereo-BRUVS sampling locations.

Location	Sites	Samples	Mean depth [range] (m)	Mean live coral cover [range] (%)
Peros Banos—Ile Diamante Lagoon	3	13	23.8 [14.0–38.0]	24.2 [0–80]
Peros Banos—Ile Diamante Reef	2	8	12.6 [8.0–18.8]	36.2 [0–50]
Peros Banos—Grouper Ground	3	12	8.8 [6.0–12.0]	65.8 [0–100]
Peros Banos—Ile de Coin lagoon	2	10	30.1 [5.4–37.9]	49.0 [20–100]
Peros Banos—Ile de Vache Marin	2	9	21.5 [14.1–31.9]	38.3 [0–80]
Salomon Atoll—Lagoon	3	15	26.8 [18.0–38.0]	38.7 [0–80]
Salomon Atoll—Reef	2	7	19.3 [12.0–23.0]	62.9 [50–80]
Victory Bank—Lagoon	2	8	28.5 [19.1–40.0]	72.5 [50–90]
Victory Bank—Reef	2	8	11.4 [9.0–20.0]	56.9 [10–90]
North Brother Island	3	12	24.7 [20.5–29.8]	20.8 [0–60]
Eagle Island	3	15	23.6 [12.8–30.0]	23.7 [0–60]
Danger Island	3	11	24.3 [17.6–27.9]	35.5 [0–80]
Sandes’ Seamount	2	10	73.1 [68.3–82.2]	0.0 [0–0]

### Processing of stereo-BRUVS video

Video samples were converted to AVI format using Xilisoft Video Converter (www.xilisoft.com) and analysed using EventMeasure (www.SeaGIS.com.au). A trained analyst processed 60 minutes of footage from one of the cameras in a stereo set (typically the left) from the time that the rig settled on the seabed. All fishes and sharks were manually identified to family, genus and species level where possible, and this information was recorded along with sample metadata (sample code, frame number and time of occurrence) and logged automatically by the software. To eliminate double counting of individuals, abundance in stereo-BRUVS samples is estimated as Max*N*, the maximum number of individuals of a given species in any single frame of video, which is a consistent and conservative measure of relative abundance [59]. EventMeasure automatically calculates Max*N* for each species based on the maximum number of concurrent identification records created for each species by the analyst as they advance through the video sample. To reduce operator bias, a second analyst checked species identifications and counts for each sample. Stereo analysis using EventMeasure then uses synchronised images from both cameras to compute lengths for each animal using a photogrammetric approach [59]. Length measurements with a root mean square (RMS) error greater than 10mm or a measurement error greater than 10% of the estimated length were rejected. Such errors typically resulted from either poor visibility or extreme orientation or range from the cameras.

In addition to the data extracted using EventMeasure, sample depth was obtained from fieldwork logs, live coral cover was estimated as the percentage of live coral (to the nearest 10%) within the camera field of view in a video still, and each sample location was classified as either reef or lagoon based on GIS data for the BMR [61]. Shark and fish biomass were calculated based on estimated lengths from the stereo-BRUVS data. Relative biomass for each species by sample was calculated as the product of a species’ Max*N* and mean individual weight, which was calculated using the species’ mean length in a sample and the length-weight relationship W = aL^b^ (W is the estimated weight; L is fork, total or standard length, with estimated fork length converted to either total or standard length as required; a and b are species-specific coefficients obtained from empirical relationships [29]). If species-specific coefficients for the length-weight equation were unavailable, those for a similarly sized congener were used, or, if necessary, for the genus or shape [62]. Where no individuals of a given species were measured in a video sample, the mean length for the species from the same site or atoll was used. Relative biomass was summed across all species to give totals for sharks and fishes for each video sample. Total fish abundance was calculated as the sum of all Max*N*s for a sample. Diversity indices for the fish community associated with each sample were calculated as Shannon-Wiener diversity (H’) [63] and Pielou evenness (J) [64] based on species abundances for fishes only. Effective species richness (ESR) was also calculated as e^H’^, a less variable measure of species richness that reduces the influence of extremely rare or transient species. The final data set comprised two variables related to the shark assemblage–species abundance (TA)–for all sharks and by species; and eight independent variables for environmental attributes and measures of the fish assemblage–depth (DEPTH), live hard coral cover (LHC), macro habitat type (TYPE–reef or lagoon), fish abundance (FISH), fish biomass (FISHBIO), fish species diversity (H), fish species evenness (J) and effective species richness (ESR). Additionally, fish biomass was disaggregated by diet/functional group based on Ruppert et al. [7] so that groups could be modelled separately as predictor variables, since studies have found reef shark diets to favour particular species groups such as planktivores [19]. Data from sample replicates were averaged for each of the 35 sites, using mean values for continuous variables and modal values for categorical variables.

### Statistical analyses

All analyses were performed using the R statistical software and additional packages. Confidence intervals (CI) of means are reported as mean ± 1.96 standard errors.

Mean abundance and the per cent of samples where present were calculated for all sharks and for each species, for the whole area and by atoll. Differences in total abundance and species composition between atolls were tested for with analysis of variance (AOV) and permutational multivariate analysis of variance (PERMANOVA) respectively. PERMANOVA was performed on a Bray-Curtis dissimilarity matrix, with empty samples removed, using the vegan package function *adonis()*. Mean depth of observation was calculated for each species. Mean fork length of grey reef sharks (the most abundant species) was calculated for each of the atolls surveyed. The hypothesis that length distributions were equal at all atolls was tested on the raw length data using the function *sm*.*density*.*compare()*. Size range at birth was taken from Robbins (2006) [65] as 54–61 cm total length (TL) at birth. This was converted to an estimate of 46–52 cm fork length (FL) at birth, based on a total length to fork length ratio of 1.173 [29]. The species growth rate during the first year was taken to be 40% [65], giving a size range for young of year of 46–73 cm FL.

Prior to linear modelling, data exploration [66] used boxplots and dot charts to identify potential outliers, and histograms and Q-Q plots were then used to assess variable normality. Log transformations were applied to the measures of shark abundance and fish biomass. Pair plots were used to assess collinearity between pairs of predictor variables (S1 Fig). Variance inflation factors (VIFs) were calculated for the continuous variables to identify redundancy and multi-collinearity among predictor variables for multiple regression, and variables with the highest VIFs were sequentially removed until all VIFs were less than four [66]. Correlation between continuous and categorical variables was assessed with conditional boxplots (S2 Fig). The final set of predictors used in model building was depth, live coral cover, and macro-habitat type for habitat variables, together with log-transformed fish biomass for low trophic level carnivores, planktivores, corallivores, detritivores and herbivores (Table 2).

**Table 2:** Dependent variables and candidate predictors used in linear models.

Dependent variables	Description
log(Total shark abundance)	Max*N* as estimated in EventMeasure
log(Grey reef shark abundance)	Max*N* as estimated in EventMeasure
*Physical predictor variables*	
Depth	Sample depth in metres
Live hard coral cover	Percentage of live hard corals as a share of visible substrate
Macro-habitat type	Reef or Lagoon
*Biological predictor variables*	
log(Low TL carnivore biomass)	log-transformed biomass, calculated from mean species length and abundance per sample as measured in EventMeasure
log(Planktivore biomass)	
log(Corallivore biomass)	
log(Detritivore biomass)	
log(Herbivore biomass)	

Generalised linear models with a Gaussian link function [67] were used to model relationships between shark abundance (all sharks, and grey reef sharks) and the predictor variables defined above, using the R function *glm()*. The n/10 ‘rule of thumb’ [68] set the maximum number of independent variable to be included in the model at three. The *step()* function was used to perform backward model selection to determine the best three variable model by evaluating the change in the model’s Aikike Information Criteria (AIC) when variables were sequentially removed. Alternative models based on the best three variables were compared with each other and a null model (intercept only) based on sample-size corrected AIC (AICc). Standardised coefficients were calculated for the best models to compare effect sizes, and partial residual plots used to visualise effects. Model fit was visualised by plotting observed against fitted values for the best models.

## Results

### Shark abundance and distribution

**Fig 2:**
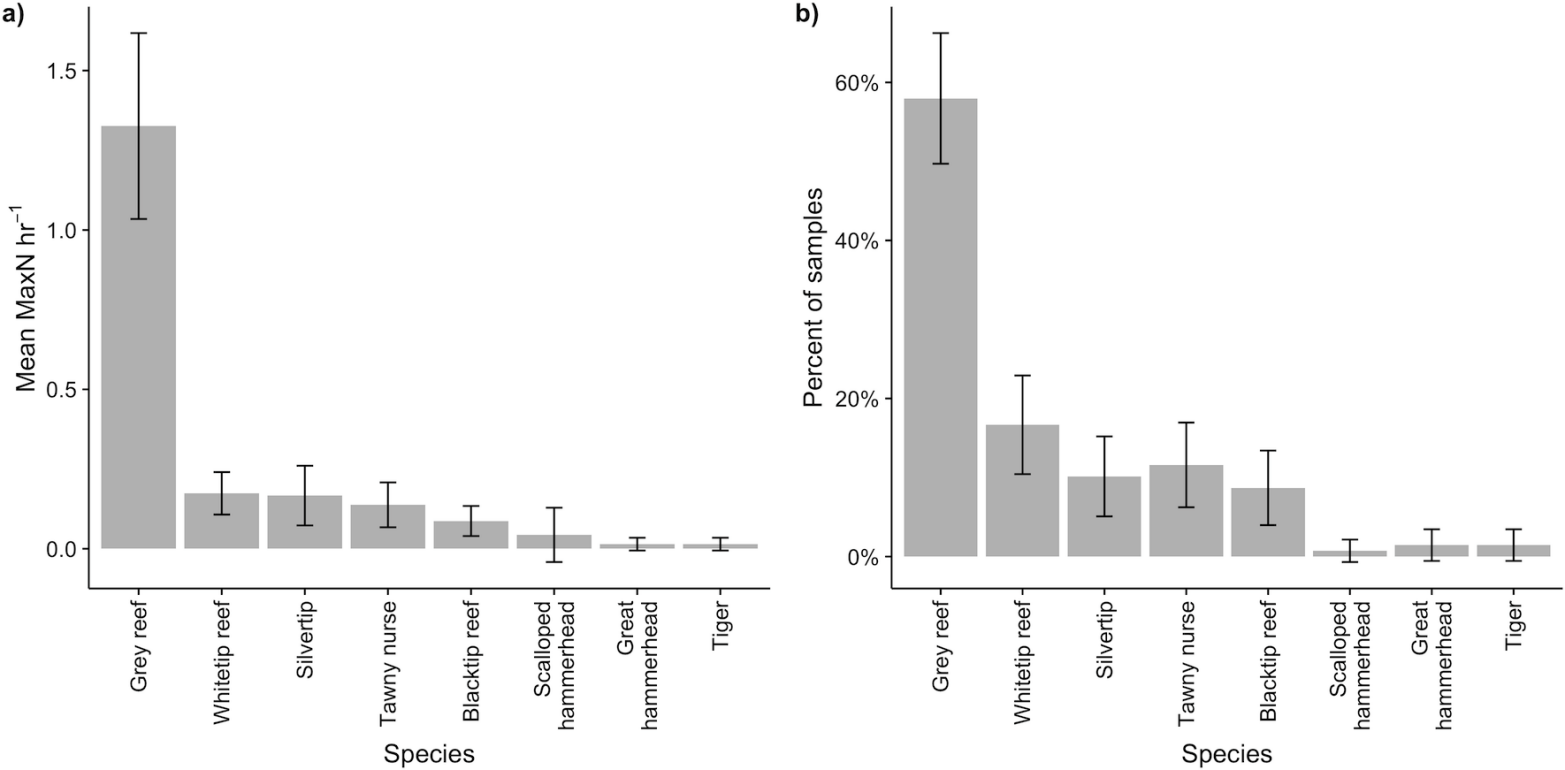
Mean abundance (Max*N* hr^-1^), and per cent of samples where present, for reef-associated sharks by species across the BMR. Error bars show 95% confidence interval.

**Fig 3:**
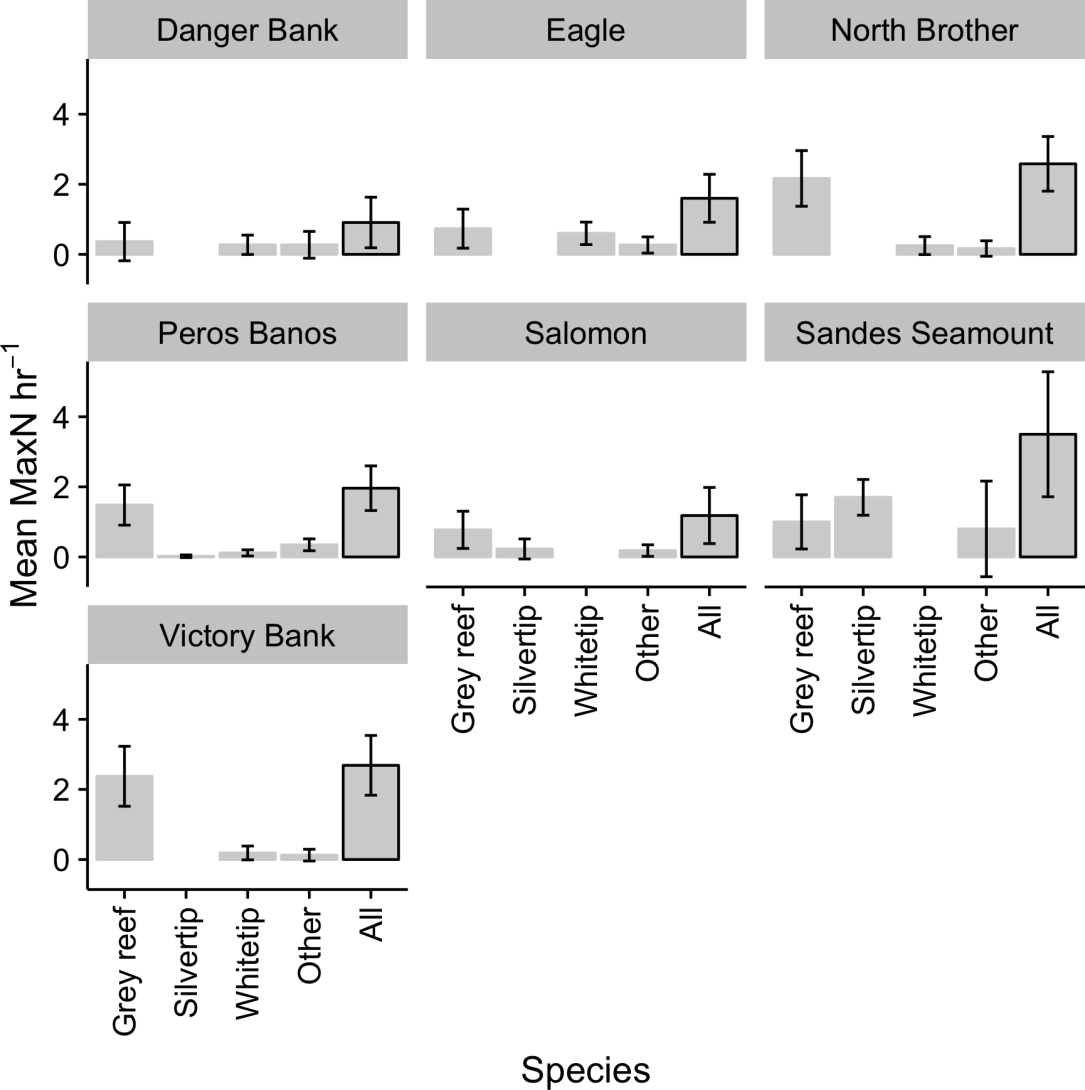
Mean abundance of reef-associated sharks at individual atolls; error bars show confidence interval of means. 'Other’ species are tawny nurse, blacktip reef, tiger, scalloped hammerhead and great hammerhead.

Mean shark abundance was 1.96 ± 0.35 hr^-1^ (n = 138). The reef-associated shark assemblage observed in the BMR comprised eight species but was dominated by grey reef sharks. These averaged 1.33 ± 0.29 individuals per hour (67% of all sharks observed) and were present on 58% of samples, compared with 0.17 ± 0.09 hr^-1^ and 17% of samples for white tip reef sharks, the next most common species (Fig 2). The third most abundant and common species was the silvertip shark (0.17 ± 0.09 hr^-1^; 10%), followed by tawny nurse (*Nebrius ferrugineus*) and blacktip reef sharks. The remaining three species were low in overall abundance and rarely observed, with tiger sharks and scalloped (*Sphyrna lewini*) and great hammerheads (*Sphyrna mokarran*) collectively totalling 10 individuals (0.07 hr^-1^) present in only 3% of samples (Fig 2). Across the BMR, sharks were most abundant at Sandes Seamount with 3.50 ± 1.78 hr^-1^, followed by Victory Bank (2.69 ± 0.85 hr^-1^) and North Brother (2.58 ± 0.78 hr^-1^). Danger Bank had the lowest abundance at 0.90 ± 0.31 hr^-1^. Grey reef sharks were the most abundant species at all locations except Sandes Seamount, where silvertips were more numerous (Fig 3). Both the overall abundance (F_[6,131]_ = 10.71, p = 0.02) and composition (F_[6,96]_ = 5.32, p = 0.001) of sharks differed significantly between atolls.

### Depth of observation

**Fig 4:**
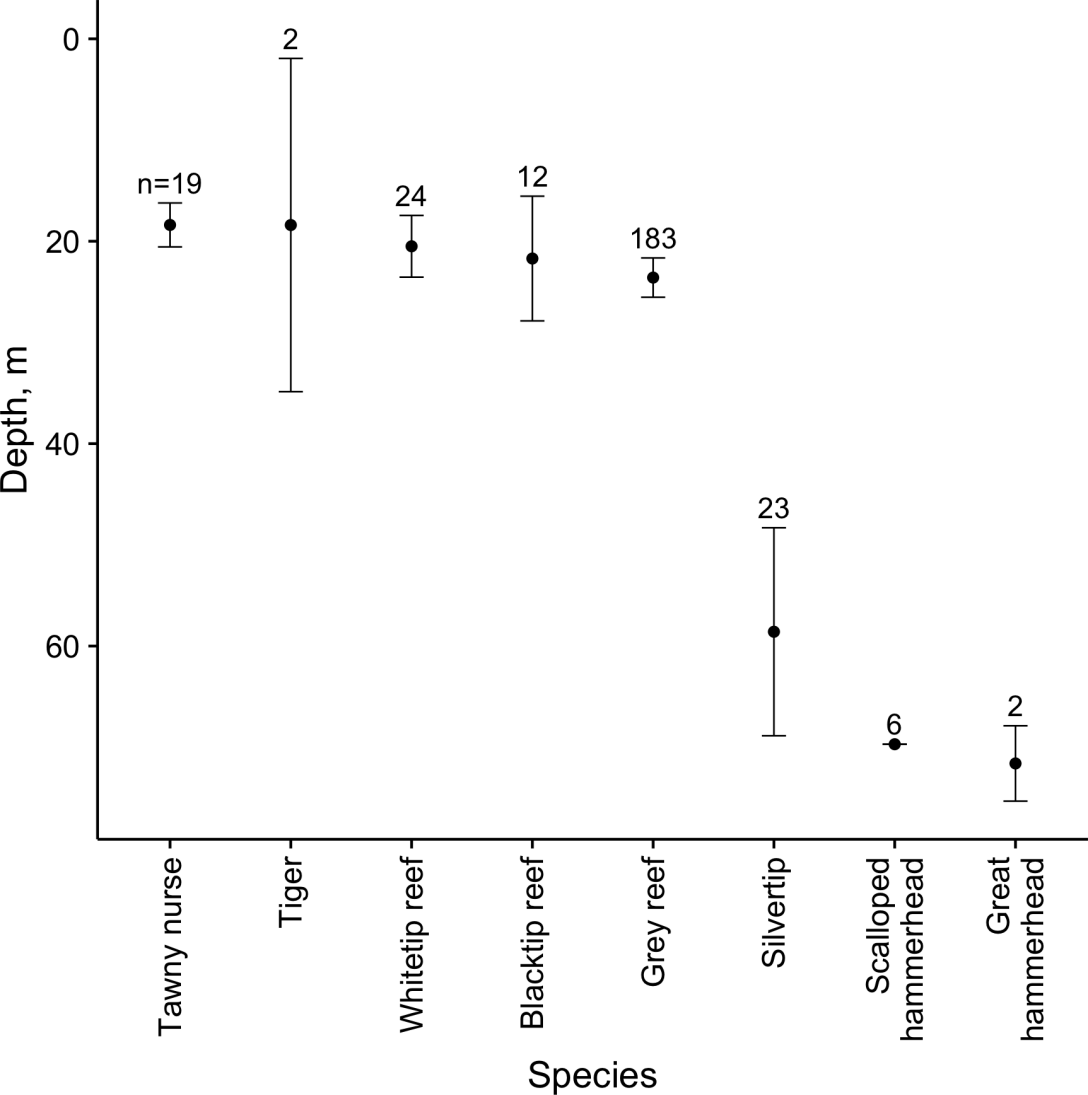
Mean depth (± 95% CI) of shark species observed on BRUVS samples in the BMR; species are ranked in order of increasing depth.

Mean depth of observation differed between species, with five species (tawny nurse, tiger, white tip reef, black tip reef and grey reef) observed predominantly on shallow sites (<40 m) and three species (silvertip, scalloped and great hammerheads) observed mainly on deeper sites. 95% confidence intervals for the mean depths indicate that the two groups occupied significantly different depth ranges (Fig 4).

### Grey reef shark lengths

**Fig 5:**
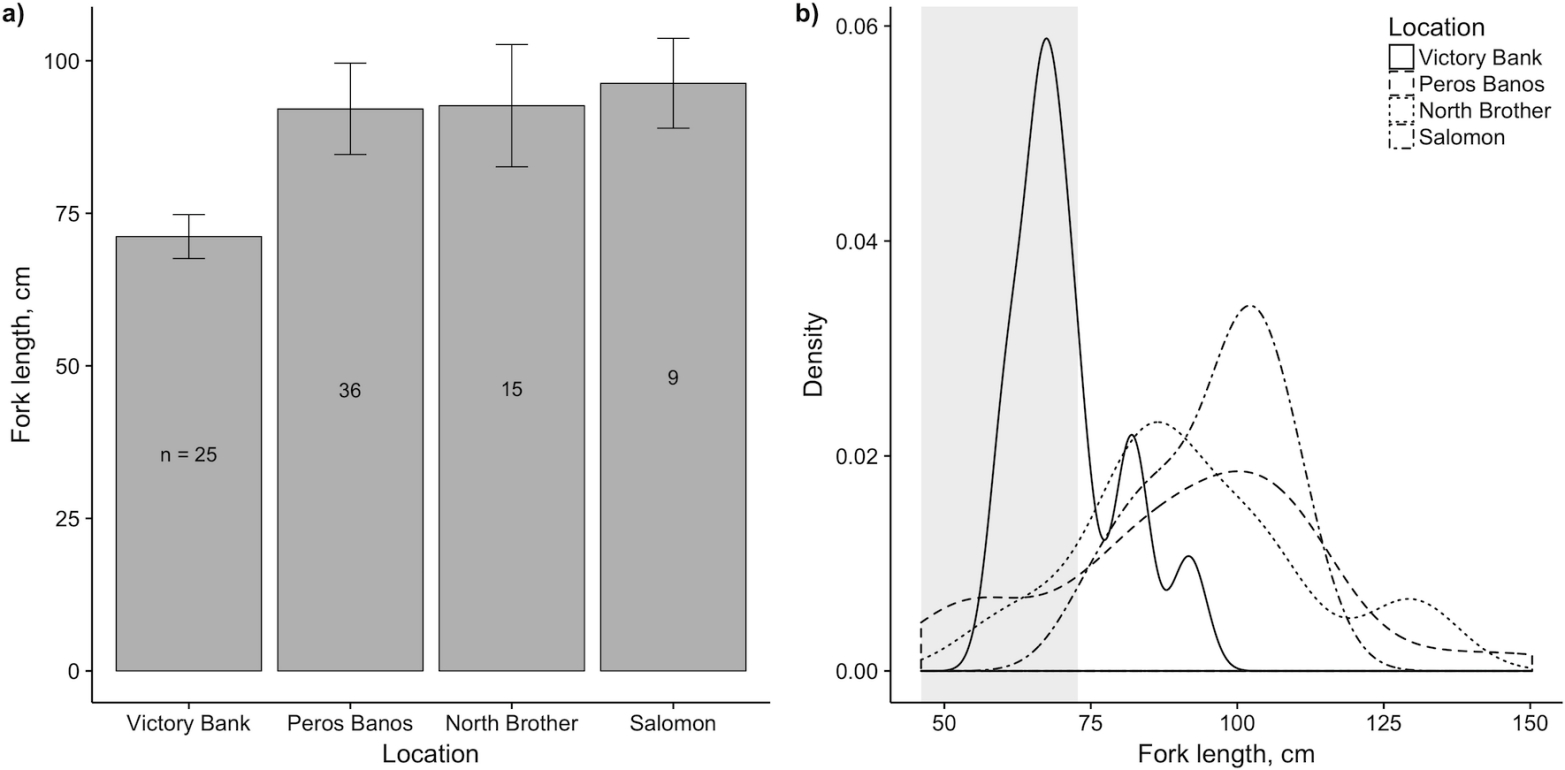
a) Mean values (± 95% CI) and b) density plots of fork lengths for grey reef sharks at Victory Bank, Peros Banhos, North Brother and Salomon reefs. Shaded region in b) indicates estimated size range of 46–73 cm FL for young of year.

The mean fork length of individual grey reef sharks differed significantly between locations where the species was sufficiently abundant for differences to be tested, with individuals at Victory Bank significantly smaller than those observed at other reefs (Fig 5A). Individuals at Victory Bank had a mean fork length of 71.2 cm, vs. 92.1, 92.6 and 96.3 cm at Peros Banhos, North Brother and Salomon reefs, respectively. The distribution of lengths at Victory Bank and the other three locations also differed significantly (p < 0.001), with lengths for the individuals at Victory Bank biased towards the size range expected for grey reef sharks during their first year (i.e. young of year, YOY; Fig 5B). 72% (18) of the sharks measured at Victory Bank were less than 73 cm FL (the upper bound of the size range estimate for YOY).

### Drivers of shark abundance

**Fig 6:**
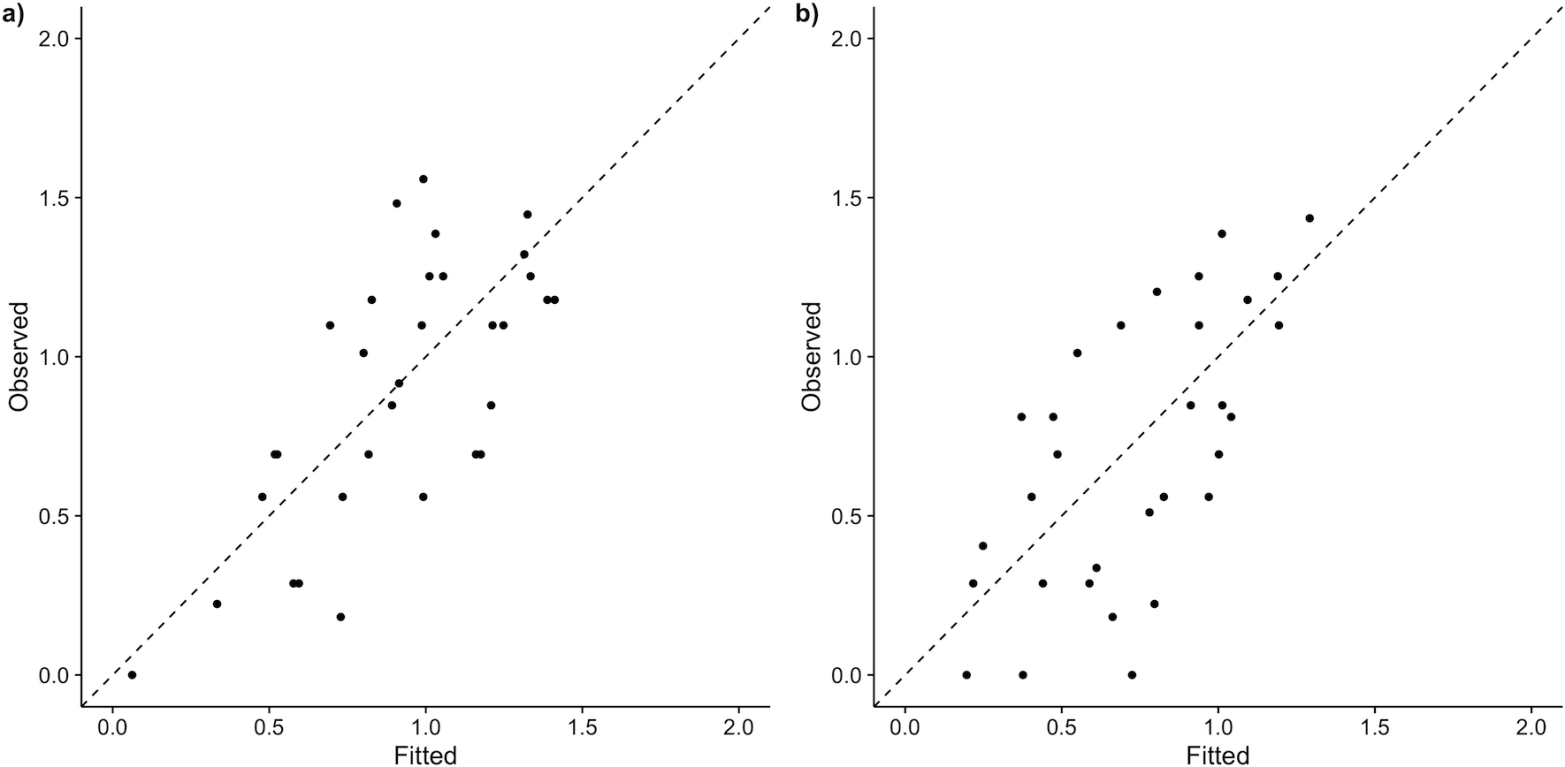
Observed vs fitted values for generalised linear models of a) log transformed total shark abundance b) log transformed abundance of grey reef sharks only.

Since Sandes Seamount was characterised by significantly greater depth (68.3–82.2m), discontinuous with the depth range of the remaining data (10-40m), the two sites at that location (Table 1) were excluded from generalised linear modelling, as they were outliers with respect to the depth variable. This left 33 sites among the remaining atolls. Following data exploration and variable selection, alternative models for total shark abundance were compared based on AICc and Aikike weight (S1 Table). The best model of total shark abundance explained 50% of the variance, with total abundance positively related to planktivore biomass, negatively correlated with the percentage of live coral cover, and higher on reef sites as opposed to lagoon sites (Table 3A). Grey reef shark abundance increased with planktivore biomass and depth, and grey reef sharks were more abundant on reef sites (Table 3B, S2 Table). Standardised model parameters for both models (Table 3) and plots of partial residuals (S3 Fig) showed planktivore biomass to have the largest effect. Plots of observed against predicted values for each model indicated that model fit for all sharks and grey reef sharks were satisfactory with respect to heteroscedascity and normality of residuals (Fig 6A and 6B).

**Table 3:** Linear regression model parameters for shark a) species richness b) abundance and c) biomass based on n = 33 sites. β = standardised model parameters.

a) log(Total shark abundance)	~ log(Planktivore biomass)+Live hard coral cover (%) + Macro-habitat type			
	Estimate	β	t	p
Intercept	-1.168	-	8.138	<0.001
log(Planktivore biomass)	0.11	0.589	3.892	<0.001
Live hard coral cover (%)	-0.857	-0.354	-2.442	0.021
Macro-habitat type (Reef = 1)	0.293	0.293	1.932	0.063
Model		R^2^_adj_ = 0.50	F_[__3__,__29__]_ = 11.48	p < 0.001
b) log(Grey reef shark abundance)	~ log(Planktivore biomass)+ Depth (m) + Macro-habitat type			
	Estimate	β	t	p
Intercept	0.05	-	0.154	0.879
log(Planktivore biomass)	0.109	0.543	3.282	0.003
Depth (m)	0.024	0.348	1.946	0.061
Macro-habitat type (Reef = 1)	0.398	0.398	2.137	0.041
Model		R^2^_adj_ = 0.41	F_[__3__,__29__]_ = 8.54	p < 0.001

## Discussion

The aim of our study was to model the regional drivers of spatial variation in shark abundance at the scale of an entire reef system, using data from a large and relatively undisturbed reef system. We modelled shark abundance and size structure against physical measures (depth, habitat quality, topography, etc.) and measures of ‘prey availability’ (biomass of fishes in lower trophic order functional groups). While grey reef sharks were the most abundant species, and were observed at all atolls, the composition of the shark assemblage varied by atoll. Sandes Seamount notably had the highest abundances of silvertip sharks, as well as two hammerhead species observed nowhere else during the study. Species clearly partitioned by depth, with some evidence suggesting spatial partitioning by size within the grey reef population. Linear modelling found that shark abundance was most strongly correlated with the biomass of planktivorous fishes. Habitat (coral cover and reef or lagoon habitat type) and depth were also significant but were weaker predictors. These results suggest that the high fish biomass recorded in the BMR [69] and the broad spectrum of habitats to be found there ranging from shallow lagoons (depth <15m) to deep seamounts (depth >70m) may be important drivers of shark abundance and diversity in the marine reserve.

The idea that mobile predators, such as sharks, respond to prey availability rather than habitat suitability has been suggested by both terrestrial and marine studies. Keim et al. [54] found that indices of lynx abundance were better correlated with evidence of prey (hare tracks) than the presence of suitable prey habitat. In marine systems, the inclusion of measures of prey abundance improves the fit of distribution models for both pelagic [55] and coastal [56] predators. On reef systems, Heupel et al. [57] also found that movement patterns of grey reef sharks in the southern Great Barrier Reef were best modelled using biological factors related to prey availability and reproduction, rather than environmental conditions. Given the mobility of large reef predators and their lower dependence on reef structures as physical refuges, particularly for obligate ram-ventilating species such as grey reef sharks, prey availability may be a more important feature of reef sites than reef structure or composition. The relative strength of fish biomass versus environmental measures in predicting the abundance of sharks must be interpreted with care, as the relative importance of biological measures may be due to their acting as proxies for environmental measures that were not measured or were measured in insufficient detail (in other words, high fish biomass may reflect ‘good’ habitat that also drives the abundance of sharks). However, the presence of this relationship in studies of other predatory taxa suggests that prey availability may be a common driver of spatial variation in predator abundance across diverse ecosystems, including coral reefs [55–57].

The composition of the shark assemblage varied significantly across the BMR, and analysis of the depth of observation of each species suggested that site depth may influence the species assemblage. The eight species observed were broadly divided into those seen only on the shallower (<40m) reef and lagoon habitats (white tip and blacktip reef sharks, tawny nurse sharks and tiger sharks), those only observed on the deepest sites (scalloped and great hammerheads), and more generalist species (grey reef and silvertip sharks). Taken together, these results suggest that a degree of vertical partitioning may exist amongst species within BMR. However, care should be taken in drawing too strong an inference from these data since the deepest site was a seamount with very different habitat characteristics to the other atolls, and was closest to the populated military base at Diego Garcia. Hammerhead shark species are highly valued by fishermen for the fin trade [70], and their absence at the atolls more distant from the locus of enforcement activity may reflect greater exposure to illegal fishing rather than their natural distribution. However, vertical partitioning as a possible driver of variation in species composition within BIOT is consistent with observations of spatial partitioning and species-specific habitat preferences found in other studies of reef sharks, including acoustic telemetry studies of white tip and grey reef sharks at Osprey Reef in the Coral Sea [38], long line surveys in the French Frigate Shoals [51] and Hawaiian Islands [40], and stereo-BRUVS studies in north west Australia [52]. The use of different depth ranges by the various species may also be one reason that depth was a significant variable in the model of grey reef shark abundance, but not for overall shark abundance: in the overall abundance model the influence of depth would have been confounded by the differing responses of individual species to that driver.

The large and significant variation in the mean size of individual grey reef sharks observed at different reefs suggests that size-based, and therefore possibly ontogenetic, spatial partitioning may be occurring in this species. The mean fork length of individuals recorded at Victory Bank was 71.2 ± 3.6 cm, significantly smaller than the mean for the other locations where grey reef sharks were abundant. This was within the estimated size range for young of year of this species, based on size at birth of 46–52 cm FL (54–61 cm TL) and 40% growth in the first year [65]. Natal philopatry by females and the consequent establishment of defined nursery areas has been documented in other carcharhinid sharks [71], including black tip reef sharks [26] and bull sharks (*Carcharhinus leucas*) [72], so a concentration of juvenile animals at a single location in the BMR may be evidence of similar behaviour among grey reef sharks. Heupel et al. [73] propose three criteria to qualify a location as a pupping site for a shark species: significantly higher abundance, use over multiple years, and evidence of increased residency. Whilst a single BRUVS survey cannot inform us about residency patterns, the data do indicate that there were both relatively high numbers of grey reef sharks in this location, and that a large proportion of them (72%) were within range of the species’ estimated size for young of year. At least 18 individuals in the sample from Victory Bank could be classified as young of year. Since grey reef shark litter size is estimated to be between 3 and 4 pups [65], this implies that at between four and six litters of sharks had been born in the location in the previous 12 months, indicating use of the site by multiple females. Furthermore, grey reef sharks caught at Victory Bank during an acoustic tagging project in March 2014, two years after the data in this study were collected, also included large numbers of juveniles (<80cm FL), some of which were observed with umbilical scars still present (DT, pers. obs.). Grey reef shark females are thought to pup every other year [74], so the 2014 observations may have followed a subsequent pupping event. Whilst the presence of juvenile sharks on BRUVS cannot confirm a pupping site per Heupel et al.’s definition, the observation of a relatively large number of juvenile grey reef sharks of a similar age from multiple litters does suggest that the site was used by multiple females to give birth during the preceding year. The fact that large numbers of grey reef sharks of a similar size were also observed two years later, consistent with a biennial breeding cycle, indicates that this may not have been an isolated event and that the location is used consistently for parturition by this species. Acoustic tagging, on-going in the BMR, may help shed further light on this question.

These results have implications for the management of sharks in the BMR and the design of MPAs more generally. Our study found shark abundance to be primarily driven by fish biomass amongst lower trophic levels and functional groups. The importance of fish biomass in predicting shark abundance suggests the necessity of ecosystem level protection, involving all species and functional groups, rather than species-specific policies, such as shark sanctuaries, which might still permit on-going depletion of prey species. A similar prey-depletion hypothesis has been put forward to explain changes in marine predator densities in the Mediterranean, where overfishing of prey species such as anchovies and sardines was thought to be responsible for reductions in near-shore encounters with dolphins, tuna and billfish [75]. Similarly, studies across a range of marine ecosystems have found that assemblages of top level predators such as sharks require both healthy environments in terms of prey availability [56], and a wide range of habitat zones to accommodate different species’ habitat preferences and to permit resource partitioning [40] and ontogenetic changes in habitat use [76,77]. Individual species or life stages preferentially use particular habitat zones or depth ranges, and, though often highly site-resident, reef shark species have been shown capable of making long movements between neighbouring reefs [39]. This implies that marine reserves that encompass a wide variety of habitats within the boundaries of the protected area may be more effective in preserving species diversity in the shark assemblage and providing the habitat niches required at different life stages. Very large MPAs such as the BMR, in contrast to more narrowly scoped or zoned protection regimes, have the additional advantage of protecting not only known and surveyed habitats but also the unknowns. At least 86 deep seamounts have been identified in BIOT, based on topographical modelling [78], and the biota had not been surveyed at any of those sites prior to the 2012 survey. Our study found large numbers of CITES listed sphyrnids at the single seamount surveyed, suggesting that the designation of the reserve to include both the shallow reef and deep water habitats of BIOT has afforded protection to previously undetected populations of vulnerable sharks.

Edgar et al. [58] found that effective MPAs shared five key features: they were large, had no fishing, and were well enforced, isolated and old. The BMR is still a relatively new reserve but it is a large, protected no-take reserve, isolated from neighbouring countries by hundreds of kilometres of deep ocean. The abundant fish biomass of its reefs, and the diverse range of habitats contained within its boundaries, from shallow reef flats in the atolls, to soft coral-covered seamounts, may be yet another reason why it is expected to remain a hub and refuge of predator biodiversity in the Indian Ocean.

## Supporting information

S1 Dataset Sample level data for all variables used in analyses. (XLSX)

S1 Document Open source GIS data acknowledgment (DOCX)

S1 Fig Pairplots of dependent and independent variables for univariate modelling. Lower panel gives Pearson’s correlation coefficient for each pair.
(TIFF)

S2 Fig Boxplots of continuous biological and habitat variables against habitat type. (TIFF)

S3 Fig Partial response plot for generalised linear models of log-transformed total shark abundance (a-c) and grey reef shark abundance (d-f).
(TIFF)

S1 Table Comparison of generalised linear model results of log-transformed shark abundance (Max*N*) in the BMR. (DOCX)

S2 Table Comparison of generalised linear model results of log-transformed grey reef shark abundance (Max*N*) in the BMR. (DOCX)

S3 Table Site variables for univariate modelling of shark indices. (DOCX)
