# Correction: Drivers of abundance and spatial distribution of reef-associated sharks in an isolated atoll reef system

**DOI:** 10.1371/journal.pone.0186560

**Published:** 2017-10-12

**Authors:** David M. Tickler, Tom B. Letessier, Heather J. Koldewey, Jessica J. Meeuwig

There is an error in affiliation 1 for authors David M. Tickler, Tom B. Letessier, and Jessica J. Meeuwig. Affiliation 1 should be: School of Biological Sciences and the Oceans Institute, University of Western Australia, 35 Stirling Highway, Crawley, Perth, WA, Australia.
