# Supplementary material for: Drivers of abundance and spatial distribution of reef-associated sharks in an isolated atoll reef system
Source: PLoS One. 2017 May 31;12(5):e0177374. doi: 10.1371/journal.pone.0177374 (PMC5451018; doi:10.1371/journal.pone.0177374)
Supplement: S1 Document — (DOCX) [file pone.0177374.s002.docx]

## S1 Open source GIS data acknowledgement

Figure 1 (map) was prepared in the open source GIS application QGIS, using the authors' own data and publicly available GIS data sets. Publicly available bathymetry data were sourced from the General Bathymetric Chart of the Oceans (GEBCO) via the British Oceanographic Data Centre (BODC). Shapefiles for the Chagos Archipelago were obtained from the Institute for Marine Remote Sensing, University of South Florida (IMaRS/USF) and Institut de Recherche pour le Développement (IRD/UR 128, Centre de Nouméa)'s Millennium Coral Reef Mapping Project (http://www.imars.usf.edu/MC/output_indian_ocean.html). Use of these data have been cited in the manuscript as Andréfouët, S., F. E. Muller-Karger, J. A. Robinson, C. J. Kranenburg, D. Torres-Pulliza, S. A. Spraggins, and B. Murch. 2005. Global assessment of modern coral reef extent and diversity for regional science and management applications: a view from space. in Y. Suzuki, T. Nakamori, M. Hidaka, H. Kayanne, B. E. Casareto, K. Nadaoka, H. Yamano, M. Tsuchiya, and K. Yamazato, editors. 10th International Coral Reef Symposium. Japanese Coral Reef Society, Okinawa, Japan. CDROM. Pages 1732-1745.
