# Supplementary figures and images for: Drivers of abundance and spatial distribution of reef-associated sharks in an isolated atoll reef system
Source: PLoS One. 2017 May 31;12(5):e0177374. doi: 10.1371/journal.pone.0177374 (PMC5451018; doi:10.1371/journal.pone.0177374)

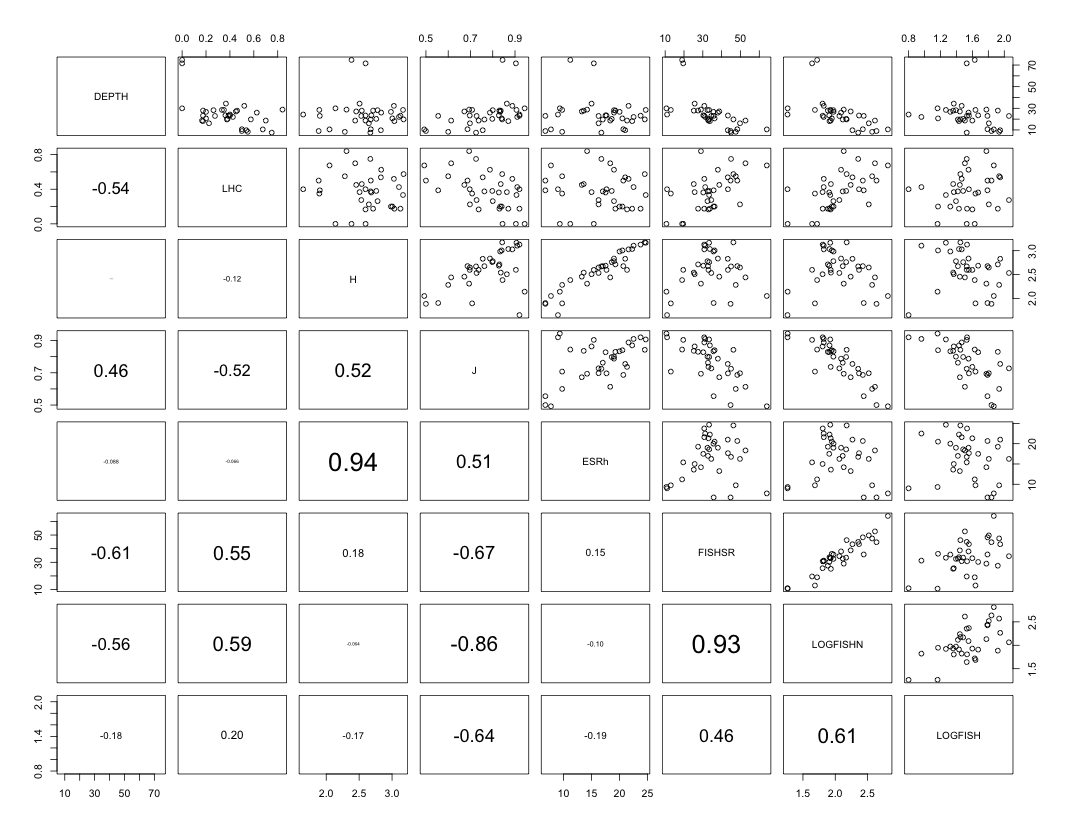

Supplement: S1 Fig — Lower panel gives Pearson’s correlation coefficient for each pair. (TIFF) [file pone.0177374.s003.tiff]

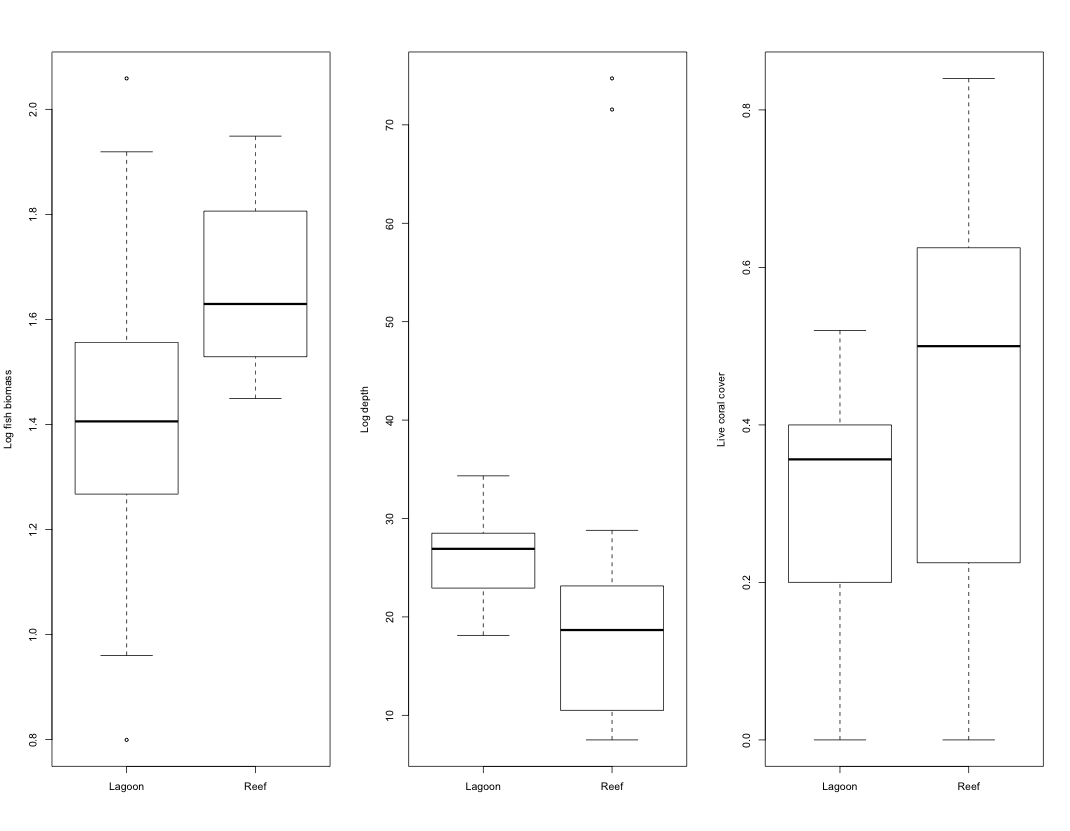

Supplement: S2 Fig — (TIFF) [file pone.0177374.s004.tiff]

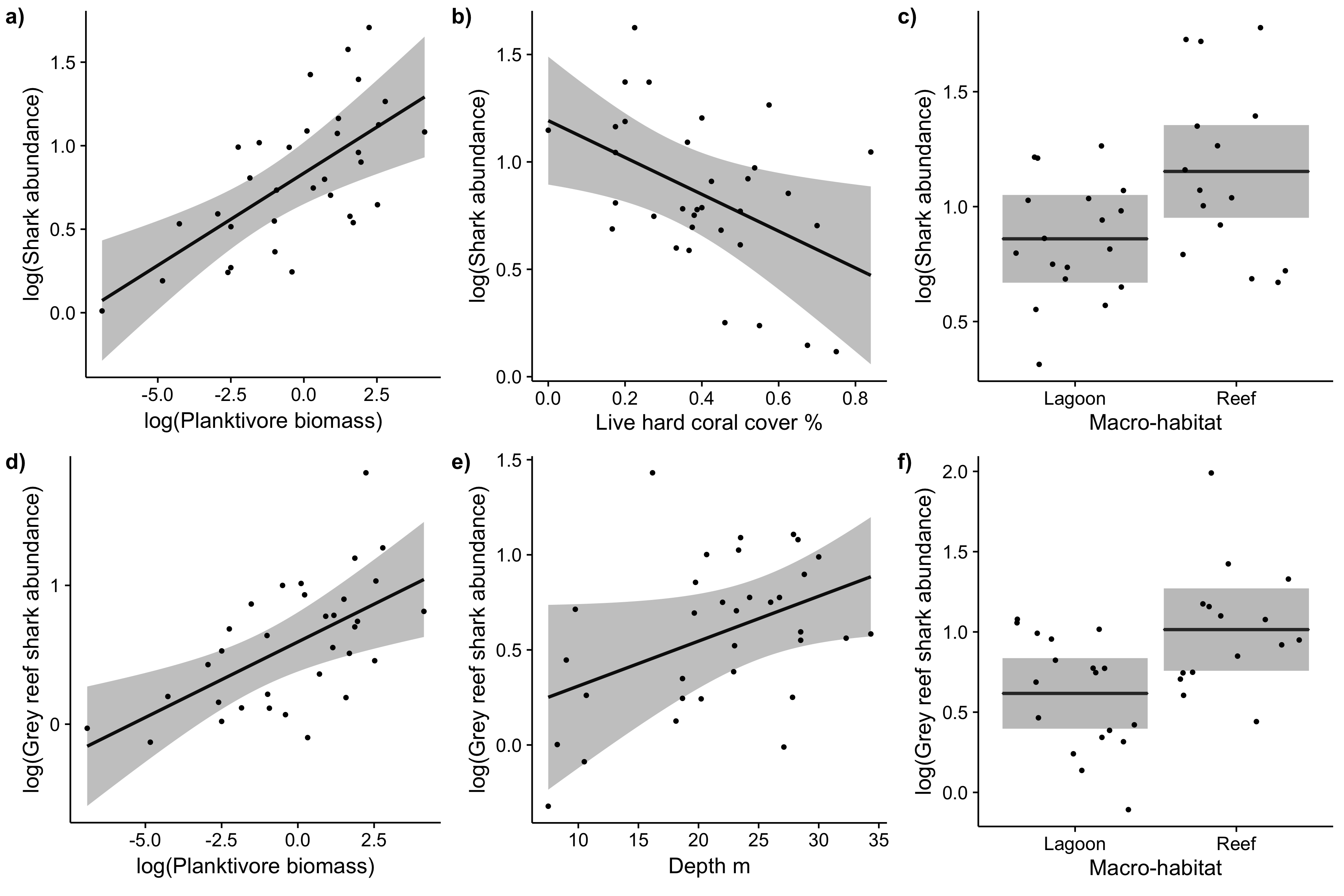

Supplement: S3 Fig — Partial response plot for generalised linear models of log-transformed total shark abundance (a-c) and grey reef shark abundance (d-f). (TIFF) [file pone.0177374.s005.tiff]
