# Supplementary material for: Drivers of abundance and spatial distribution of reef-associated sharks in an isolated atoll reef system
Source: PLoS One. 2017 May 31;12(5):e0177374. doi: 10.1371/journal.pone.0177374 (PMC5451018; doi:10.1371/journal.pone.0177374)
Supplement: S1 Table — (DOCX) [file pone.0177374.s006.docx]

S1 Table: Comparison of generalised linear model results of log-transformed shark abundance (Max*N*) in the BMR. Predictor variables used: Log-transformed Planktivore biomass, live hard coral cover and macrohabitat type (reef or lagoon). Models are ranked by increasing Akaike's information criterion corrected for sample size (AICc); the null model: log(Shark abundance) ~ 1 is shown for reference. Delta AICc (ΔAICc) and Aikike weight (wAICc) are included for ease of model comparison.

|  | df | LL | AICc | ΔAICc | wAICc |
| --- | --- | --- | --- | --- | --- |
| log(Planktivore biomass) + Live hard coral cover + Macrohabitat | 29 | -8.00 | 28.22 | 0.0 | 0.46 |
| log(Planktivore biomass) + Live hard coral cover | 30 | -10.00 | 29.42 | 1.2 | 0.25 |
| log(Planktivore biomass) | 31 | -11.57 | 29.97 | 1.7 | 0.19 |
| log(Planktivore biomass) + Macrohabitat | 30 | -11.09 | 31.60 | 3.4 | 0.09 |
| Live hard coral cover + Macrohabitat | 30 | -14.94 | 39.30 | 11.1 | 0.00 |
| Macrohabitat | 31 | -16.75 | 40.33 | 12.1 | 0.00 |
| Live hard coral cover | 32 | -20.92 | 46.24 | 18.0 | 0.00 |
| Null model | 31 | -20.92 | 48.66 | 20.4 | 0.00 |
