# Supplementary material for: Drivers of abundance and spatial distribution of reef-associated sharks in an isolated atoll reef system
Source: PLoS One. 2017 May 31;12(5):e0177374. doi: 10.1371/journal.pone.0177374 (PMC5451018; doi:10.1371/journal.pone.0177374)
Supplement: S2 Table — (DOCX) [file pone.0177374.s007.docx]

S2 Table: Comparison of generalised linear model results of log-transformed grey reef shark abundance (Max*N*) in the BMR. Predictor variables used: Log-transformed Planktivore biomass, depth and macrohabitat type (reef or lagoon). Models are ranked by increasing Akaike's information criterion corrected for sample size (AICc); the null model: log(Shark abundance) ~ 1 is shown for reference. Delta AICc (ΔAICc) and Aikike weight (wAICc) are included for ease of model comparison.

|  | df | LL | AICc | dAICc | wAICc |
| --- | --- | --- | --- | --- | --- |
| log(Planktivore biomass) + Depth + Macrohabitat | 29 | -12.67 | 37.55 | 0.0 | 0.36 |
| log(Planktivore biomass) | 31 | -15.55 | 37.93 | 0.4 | 0.30 |
| log(Planktivore biomass) + Macrohabitat | 30 | -14.69 | 38.81 | 1.3 | 0.19 |
| log(Planktivore biomass) + Depth | 30 | -15.08 | 39.59 | 2.0 | 0.13 |
| Macrohabitat | 31 | -18.66 | 44.15 | 6.6 | 0.01 |
| Depth + Macrohabitat | 30 | -17.88 | 45.19 | 7.6 | 0.01 |
| Null model | 32 | -23.11 | 50.63 | 13.1 | 0.00 |
| Depth | 31 | -22.65 | 52.13 | 14.6 | 0.00 |
