# Supplementary material for: Drivers of abundance and spatial distribution of reef-associated sharks in an isolated atoll reef system
Source: PLoS One. 2017 May 31;12(5):e0177374. doi: 10.1371/journal.pone.0177374 (PMC5451018; doi:10.1371/journal.pone.0177374)
Supplement: S3 Table — (DOCX) [file pone.0177374.s008.docx]

S3 Table: Site variables for univariate modelling of shark indices

| Site | Location | N | TA  (hr^-1^) | DEPTH  (m) | LHC | TYPE | LOGFISHBIO  (kghr^-1^) | LOGFISHN  (hr^-1^) | H  (hr^-1^) | J  (hr^-1^) | ESRh  (hr^-1^) | CAR2 | COR | DET | HER | PLA |
| --- | --- | --- | --- | --- | --- | --- | --- | --- | --- | --- | --- | --- | --- | --- | --- | --- |
| DA01 | Danger Bank | 4 | 0.75 | 19.65 | 0.38 | Lagoon | 1.42 | 2.12 | 2.68 | 0.76 | 17.00 | 2.62 | -1.52 | 0.66 | 0.37 | -1.01 |
| DA02 | Danger Bank | 3 | 1.00 | 26.73 | 0.20 | Lagoon | 1.32 | 1.97 | 2.98 | 0.83 | 20.00 | 2.35 | -1.36 | 0.45 | 0.96 | -4.26 |
| DA03 | Danger Bank | 4 | 1.00 | 27.10 | 0.45 | Lagoon | 1.44 | 2.24 | 2.46 | 0.67 | 13.25 | 2.50 | -0.84 | 1.19 | 1.40 | 0.32 |
| EA01 | Eagle Island | 4 | 2.25 | 20.68 | 0.20 | Lagoon | 1.17 | 1.95 | 3.01 | 0.84 | 20.50 | 1.65 | -2.64 | 1.06 | 0.00 | -1.53 |
| EA02 | Eagle Island | 3 | 0.33 | 28.50 | 0.33 | Lagoon | 1.27 | 1.92 | 3.17 | 0.91 | 24.67 | 2.28 | -1.88 | -0.09 | -0.32 | -2.60 |
| EA03 | Eagle Island | 4 | 2.00 | 28.28 | 0.26 | Lagoon | 1.39 | 1.97 | 2.78 | 0.80 | 19.00 | 2.78 | -1.45 | 0.44 | -0.66 | -2.25 |
| EA04 | Eagle Island | 4 | 1.50 | 18.13 | 0.18 | Lagoon | 1.43 | 1.91 | 3.04 | 0.87 | 22.25 | 2.33 | -1.91 | 1.14 | 0.18 | -0.93 |
| NB01 | North Brother | 4 | 2.25 | 23.15 | 0.18 | Reef | 1.53 | 1.81 | 3.13 | 0.92 | 23.75 | 2.36 | -3.42 | 0.13 | -0.69 | 0.91 |
| NB02 | North Brother | 4 | 2.25 | 22.93 | 0.28 | Lagoon | 2.06 | 2.06 | 2.53 | 0.73 | 16.25 | 2.45 | -1.75 | 1.84 | 0.80 | 4.13 |
| NB03 | North Brother | 4 | 3.25 | 27.90 | 0.18 | Lagoon | 1.92 | 1.89 | 2.71 | 0.83 | 19.25 | 2.12 | -2.13 | 0.66 | -0.63 | 2.79 |
| PB01 | Peros Banhos - Ile Vache Marine W | 5 | 2.00 | 20.22 | 0.38 | Reef | 1.48 | 2.17 | 2.76 | 0.80 | 18.60 | 1.89 | -1.65 | 1.36 | 0.54 | 0.71 |
| PB02 | Peros Banhos - Ile Vache Marine W | 4 | 2.50 | 23.00 | 0.39 | Reef | 1.79 | 2.44 | 1.90 | 0.56 | 6.75 | 2.61 | -2.24 | 1.15 | 1.04 | 1.87 |
| PB03 | Peros Banhos - Ile du Coin | 5 | 0.20 | 27.82 | 0.46 | Lagoon | 1.36 | 1.93 | 2.54 | 0.84 | 13.60 | 2.31 | -1.12 | -2.58 | 1.34 | -0.41 |
| PB04 | Peros Banhos - Ile du Coin | 5 | 1.00 | 32.28 | 0.52 | Lagoon | 1.47 | 1.82 | 3.03 | 0.89 | 21.60 | 2.59 | -1.16 | -2.28 | 1.70 | -1.85 |
| PB05 | Peros Banhos - Ile Vache Marine E | 4 | 1.00 | 10.50 | 0.68 | Reef | 1.87 | 2.81 | 2.05 | 0.49 | 7.75 | 3.35 | -0.33 | 0.72 | 2.44 | 2.52 |
| PB06 | Peros Banhos - Ile Vache Marine E | 4 | 1.00 | 8.25 | 0.55 | Reef | 1.94 | 2.57 | 2.28 | 0.60 | 9.75 | 2.90 | -1.19 | -0.68 | 1.39 | 1.69 |
| PB07 | Peros Banhos - Ile Vache Marine E | 4 | 0.75 | 7.50 | 0.75 | Reef | 1.53 | 2.35 | 2.67 | 0.73 | 16.75 | 2.69 | -0.98 | 0.03 | 1.50 | 1.58 |
| PB08 | Peros Banhos - Ile Diamante Inner | 4 | 1.75 | 23.50 | 0.36 | Lagoon | 1.67 | 1.91 | 2.69 | 0.83 | 17.50 | 2.83 | -0.87 | -0.02 | 0.18 | -0.50 |
| PB09 | Peros Banhos - Ile Diamante Inner | 3 | 3.00 | 23.33 | 0.40 | Lagoon | 1.56 | 2.37 | 2.60 | 0.70 | 17.67 | 2.15 | -1.20 | -0.47 | 1.34 | 1.87 |
| PB10 | Peros Banhos - Ile Diamante Inner | 3 | 1.33 | 18.67 | 0.17 | Reef | 1.60 | 1.93 | 2.60 | 0.74 | 21.33 | 2.67 | -1.76 | -3.29 | 0.87 | -0.99 |
| PB11 | Peros Banhos - Ile Diamante Outer | 3 | 1.33 | 30.00 | 0.00 | Lagoon | 1.16 | 1.26 | 2.14 | 0.94 | 9.33 | 2.19 | -2.79 | -1.19 | -6.91 | -2.50 |
| PB12 | Peros Banhos - Ile Diamante Outer | 4 | 2.75 | 9.00 | 0.50 | Reef | 1.84 | 2.64 | 1.89 | 0.50 | 6.75 | 2.93 | -1.25 | -0.56 | 1.50 | 2.56 |
| SL01 | Salomon - Lagoon | 4 | 0.00 | 24.25 | 0.40 | Lagoon | 0.80 | 1.26 | 1.65 | 0.92 | 9.00 | 2.97 | -0.85 | -0.12 | 1.79 | 2.23 |
| SL02 | Salomon - Lagoon | 4 | 0.25 | 28.50 | 0.35 | Lagoon | 1.64 | 1.69 | 1.90 | 0.71 | 9.75 | -1.75 | -1.62 | -1.97 | -1.21 | -6.91 |
| SL03 | Salomon - Lagoon | 4 | 0.75 | 22.00 | 0.43 | Lagoon | 0.96 | 1.82 | 3.11 | 0.91 | 22.50 | 1.12 | -1.91 | -1.17 | 0.45 | -4.84 |
| SL04 | Salomon - Reef | 4 | 3.75 | 19.75 | 0.58 | Reef | 1.45 | 2.18 | 3.18 | 0.84 | 24.50 | 0.34 | -0.52 | -0.57 | -0.35 | -2.95 |
| SL05 | Salomon - Lagoon | 3 | 0.33 | 34.33 | 0.37 | Lagoon | 1.37 | 1.80 | 2.51 | 0.86 | 15.00 | 2.54 | -1.04 | -0.91 | 0.64 | 0.22 |
| SL06 | Salomon - Reef | 3 | 2.00 | 18.67 | 0.70 | Reef | 1.51 | 2.61 | 2.44 | 0.61 | 18.33 | 1.82 | -1.09 | -1.32 | 0.87 | -2.50 |
| SS01 | Sandes' Seamount Outer | 5 | 2.80 | 71.56 | 0.00 | Reef | 1.53 | 1.64 | 2.60 | 0.90 | 15.40 | 2.63 | -0.45 | -1.53 | 1.73 | 1.15 |
| SS02 | Sandes' Seamount Inner | 5 | 4.20 | 74.72 | 0.00 | Reef | 1.63 | 1.72 | 2.38 | 0.84 | 11.20 | 2.08 | -6.91 | 0.95 | -0.15 | 1.98 |
| VB01 | Victory Bank Rim | 4 | 2.50 | 9.75 | 0.54 | Reef | 1.95 | 2.27 | 2.83 | 0.76 | 21.00 | 2.63 | -6.91 | 0.62 | 0.09 | 2.88 |
| VB02 | Victory Bank Rim | 3 | 2.00 | 10.67 | 0.50 | Reef | 1.79 | 2.43 | 2.68 | 0.69 | 20.67 | 3.90 | -0.93 | 0.73 | 2.71 | 0.11 |
| VB03 | Victory Bank Lagoon | 5 | 3.40 | 28.80 | 0.84 | Reef | 1.78 | 2.13 | 2.31 | 0.69 | 14.20 | 2.36 | -1.31 | -0.62 | 3.13 | 1.96 |
| VB04 | Victory Bank Lagoon | 4 | 2.50 | 26.00 | 0.63 | Reef | 1.55 | 2.09 | 2.84 | 0.79 | 19.00 | 1.74 | -1.54 | -0.01 | 1.05 | 1.51 |
